# Supplementary material for: Evolution of Dosage-Sensitive Genes by Tissue-Restricted Expression Changes
Source: Genome Biol Evol. 2025 Aug 5;17(8):evaf132. doi: 10.1093/gbe/evaf132 (PMC12321322; doi:10.1093/gbe/evaf132)
Supplement: evaf132_Supplementary_Data [file evaf132_supplementary_data.pdf]

Supporting Information: Evolution of dosage-sensitive genes by tissue-restricted expression changes

Alan M. Rice[1,2], Yuanshuo Li[1], Pauric Donnelly[1], and Aoife McLysaght[1,*]

[1]*Smurfit Institute of Genetics, Trinity College Dublin, Dublin 2, Ireland*

[2]*Milner Centre for Evolution, Department of Life Sciences, University of Bath, Bath, BA2 7AY, UK*

[*]*Correspondence to aoife.mclysaght@tcd.ie*

CNV, ohnolog, haploinsufficient, CNVR and CCN genes as proxies for dosage sensitive genes

Table S1. Logistic regression model

	Dependent variable: Affected by eQTL (Y/N)		
	Coefficient (95% CI)	Odds ratio (95% CI)	P-value
Expression level	−0.0001 (−0.0002, −0.0001)	0.9999 (0.9998, 0.9999)	0.0003
Duplication status - singleton	0.3449 (0.3305, 0.3594)	1.4119 (1.3916, 1.4325)	$< 2.2 \times 10^{-16}$
Duplication status - SSD	0.3231 (0.3090, 0.3373)	1.3815 (1.3621, 1.4011)	$< 2.2 \times 10^{-16}$
Expression level:singleton	0.0002 (0.0001, 0.0003)	1.0002 (1.0001, 1.0003)	4.45×10^{-6}
Expression level:SSD	0.0001 (0.0001, 0.0002)	1.0001 (1.0001, 1.0002)	0.0003
Constant	−1.7029 (−1.7134, −1.6925)	0.1822 (0.1803, 0.1841)	$< 2.2 \times 10^{-16}$
Observations	790,141 (14,834-18,589 genes in 48 tissues)		

Dosage-sensitive genes are affected by fewer eQTLs

The maintext analyses mainly concern proportions of tissues and eQTLs, however the absolute number of eQTLs affecting a dosage-sensitive gene could also be reduced if

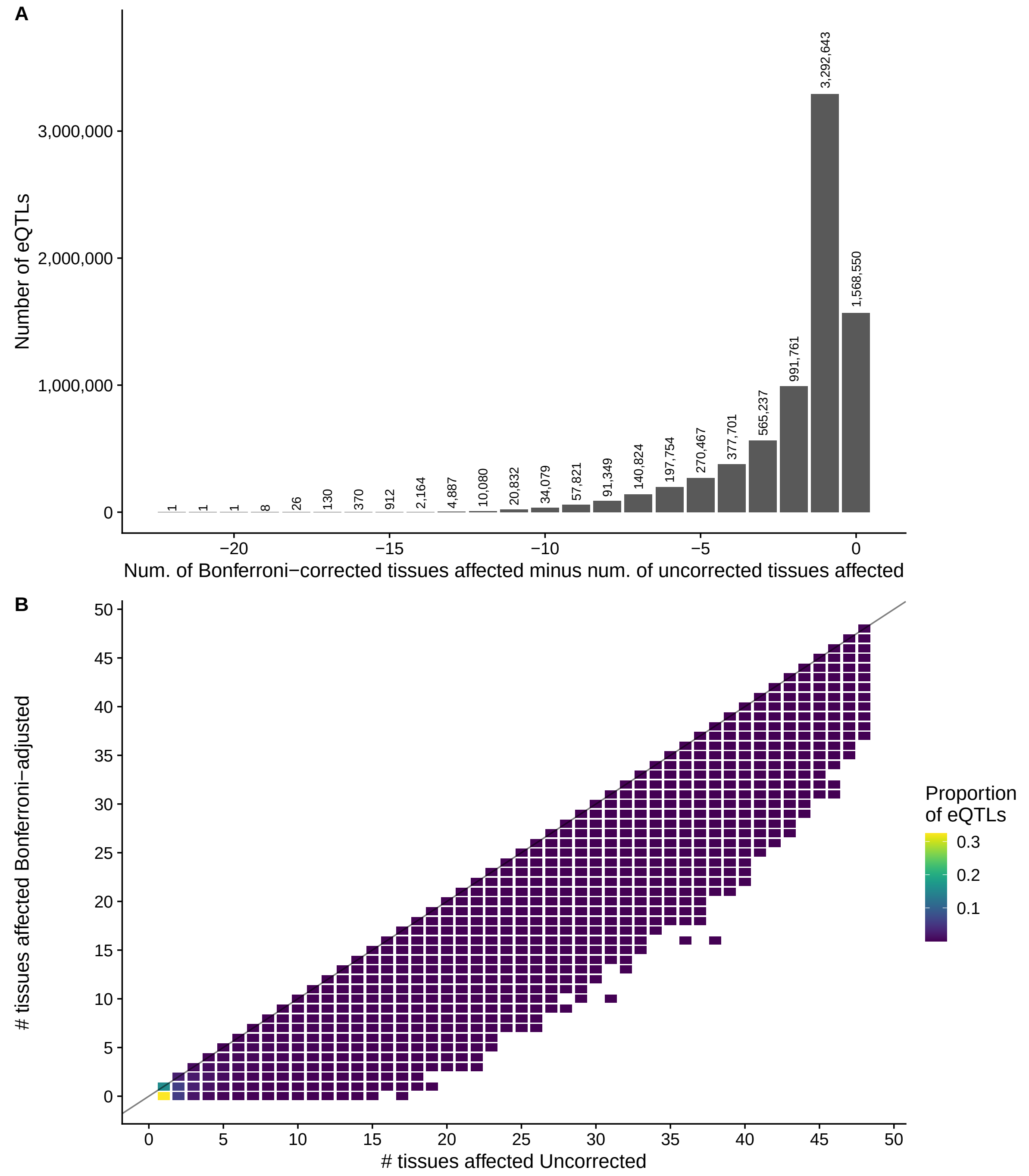

Figure S1. Effect of Bonferroni-correction on the number of tissues affected by each eQTL in the GTEx v7 single tissue eQTL data .
A Difference in the number of tissues affected by each eQTL between the uncorrected and Bonferroni-corrected GTEx v7 eQTL datasets. The x-axis represents the difference (Bonferroni-corrected tissues – Uncorrected tissues), and the y-axis represents the number of eQTLs.. **B** Heatmap showing the proportion of eQTLs affecting a given number of tissues both the uncorrected and Bonferroni-corrected datasets. The x-axis is the number of tissues affected in the uncorrected dataset, and the y-axis represents the number of tissues affected in the Bonferroni-corrected dataset. The colour intensity shows the proportion of eQTLs, with darker colours representing a lower proportion of eQTLs and brighter colours representing a higher proportion.

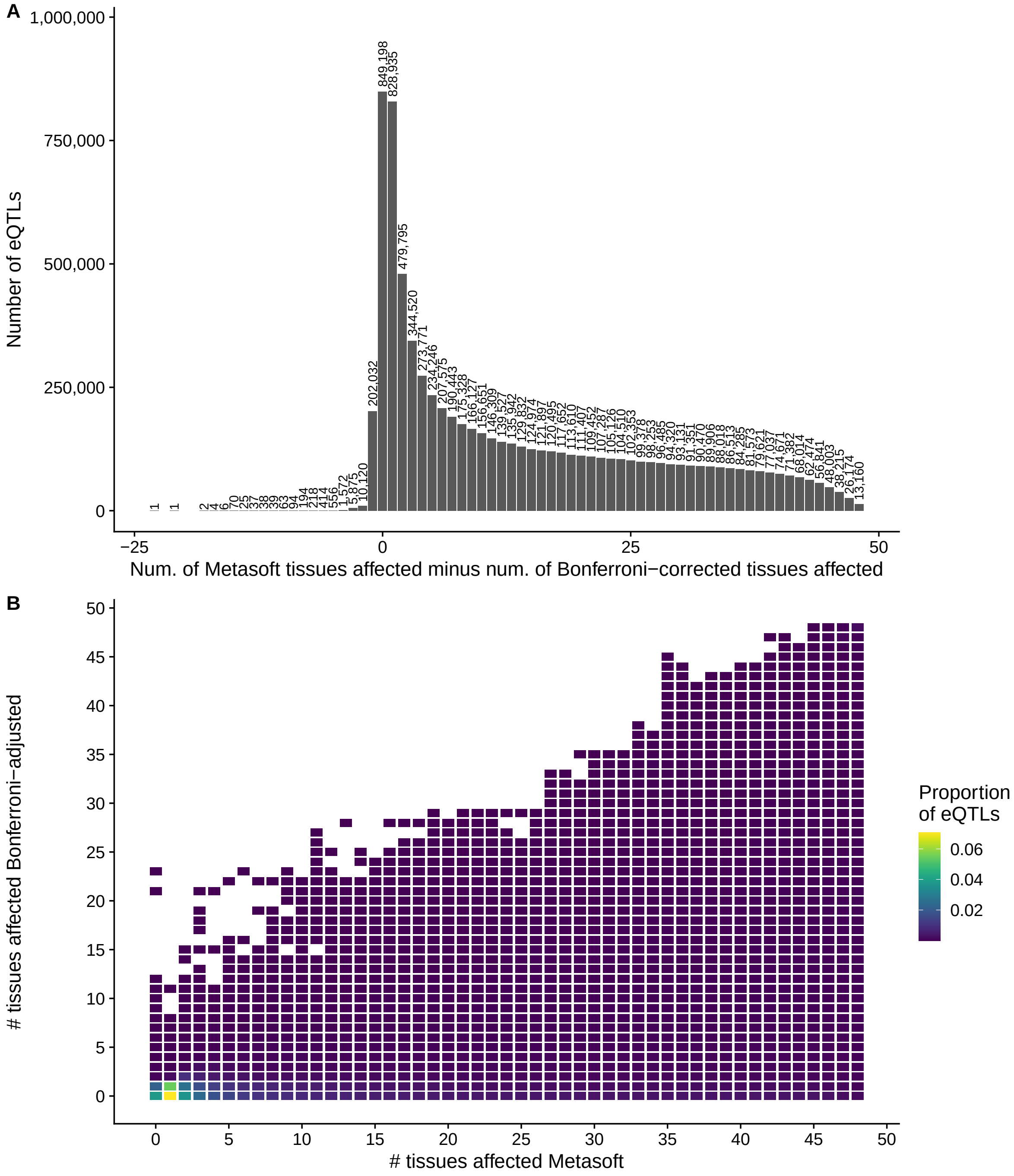

Figure S2. Effect of Metasoft processing on the number of tissues affected by each eQTL in the GTEx v7 dataset.
A Difference in the number of tissues affected by each eQTL between the Metasoft GTEx v7 eQTL dataset and Bonferroni-corrected dataset. The x-axis represents the difference (Metasoft tissues – Bonferroni tissues), and the y-axis represents the number of eQTLs. **B** Heatmap showing the proportion of eQTLs affecting a given number of tissues in both the Metasoft and Bonferroni-corrected datasets. The x-axis is the number of tissues affected in the Metasoft dataset, and the y-axis represents the number of tissues affected in the Bonferroni-corrected dataset. The colour intensity shows the proportion of eQTLs, with darker colours representing a lower proportion of eQTLs and brighter colours representing a higher proportion.

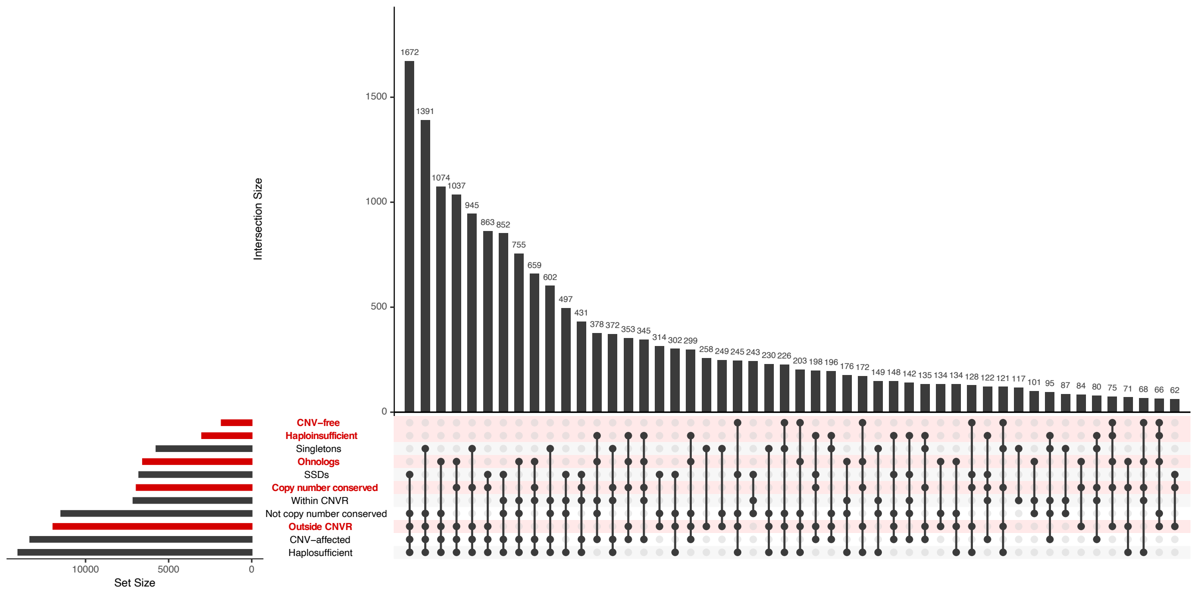

Figure S3. Overlap between different gene sets used in these analyses. This bar chart and UpSet plot shows the number of genes overlapping between different gene sets used in this study. The gene sets include: genes found to be affected by CNVs in the Exome Aggregation Consortium (ExAC) dataset and those not affected (CNV-free), genes within and outside copy number variable regions (CNVRs) of the Zarrei et al. CNV map, genes identified as singletons, small-scale duplicates (SSDs), and ohnologs (genes retained after whole-genome duplication), genes that are haplosufficient and haploinsufficient genes (pLI >0.9), and mammalian copy number conserved (CCN) genes (genes with one-to-one orthology in 13 mammalian genomes) and genes that are not conserved. Gene sets that are broadly expected to be dosage sensitive are highlighted in red. The top 50 overlaps are shown here.

Table S2. Proportions of expressed tissues affected by eQTLs for eQTL-affected genes. P-values for Mann-Whitney U tests are Bonferroni-corrected for multiple tests.

			Bonferroni-corrected eQTLs		Metasoft eQTLs	
		n	Median proportion affected tissues	P-value	Median proportion affected tissues	P-value
Zarrei et al. CNV map	Genes in CNVR	7,124	14.6%	$< 1 \times 10^{-16}$	87.5%	$< 1 \times 10^{-16}$
	Genes outside CNVRs	11,943	12.5%		81.3%	
ExAC CNV genes	CNV-affected genes	13,337	12.5%	4.3×10^{-11}	85.1%	$< 1 \times 10^{-16}$
	CNV-free genes	1,813	10.4%		68.8%	
Duplication status	Ohnologs	6,550	12.0%	1×10^{-16}	72.9%	1×10^{-16}
	Small-scale duplications (SSDs)	6,777	14.6%		87.5%	
	Singletons	5,740	14.6%		91.7%	
Conserved copy number genes	Conserved genes	6,932	12.5%	2.1×10^{-12}	81.2%	2.8×10^{-8}
	Not conserved	11,470	14.6%		85.4%	
Haploinsufficiency	Haploinsufficient genes	2,992	8.3%	$< 1 \times 10^{-16}$	66.7%	$< 1 \times 10^{-16}$
	Other genes	14,053	14.6%		87.5%	

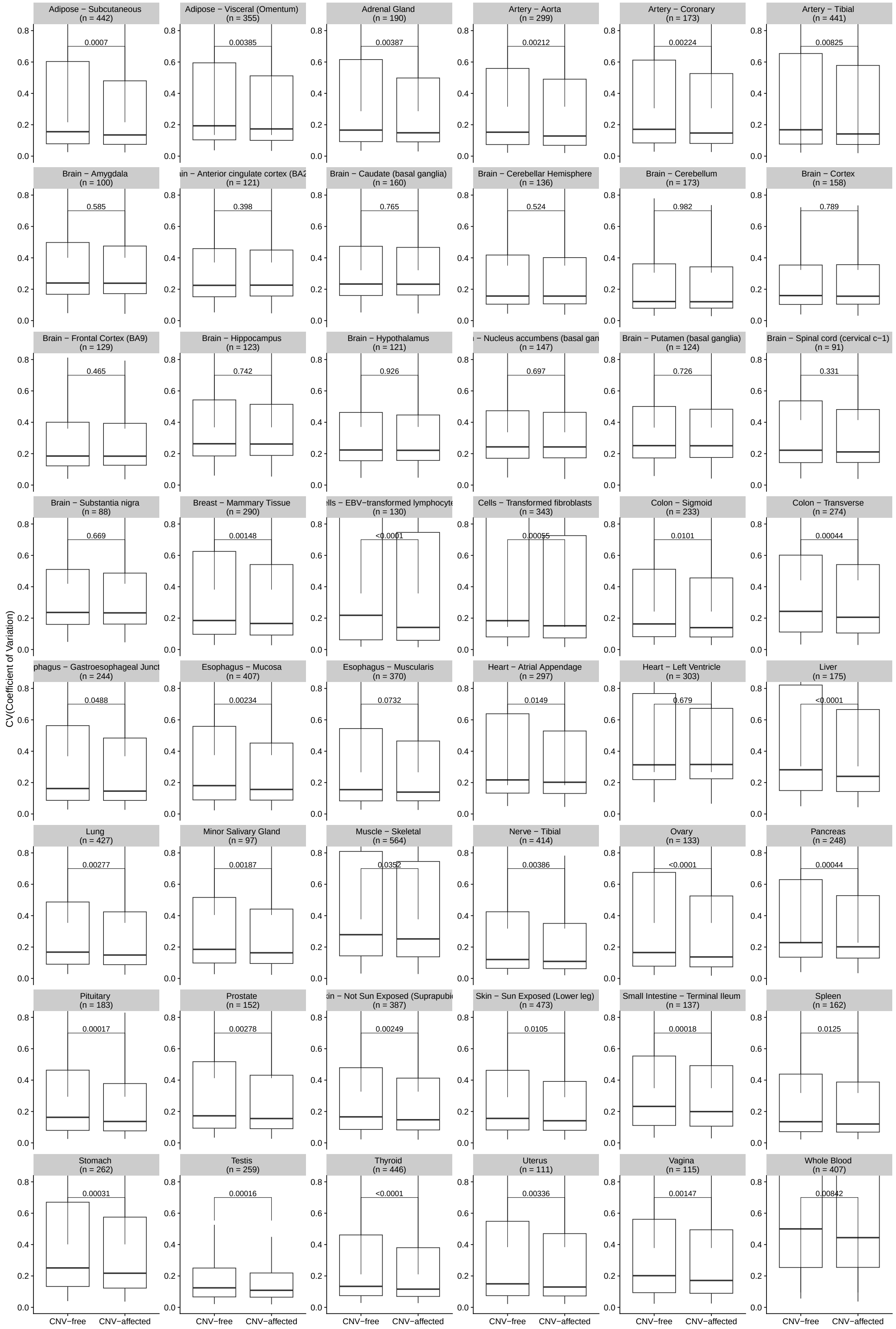

Figure S4. Coefficient of variation (CV) for CNV-free and CNV-affected genes. P-values from Wilcoxon rank-sum tests are Benjamini-Hochberg corrected for multiple comparisons across tissues. N indicates the number of individuals per tissue. The same applies to Supplementary Figures S2–S6.

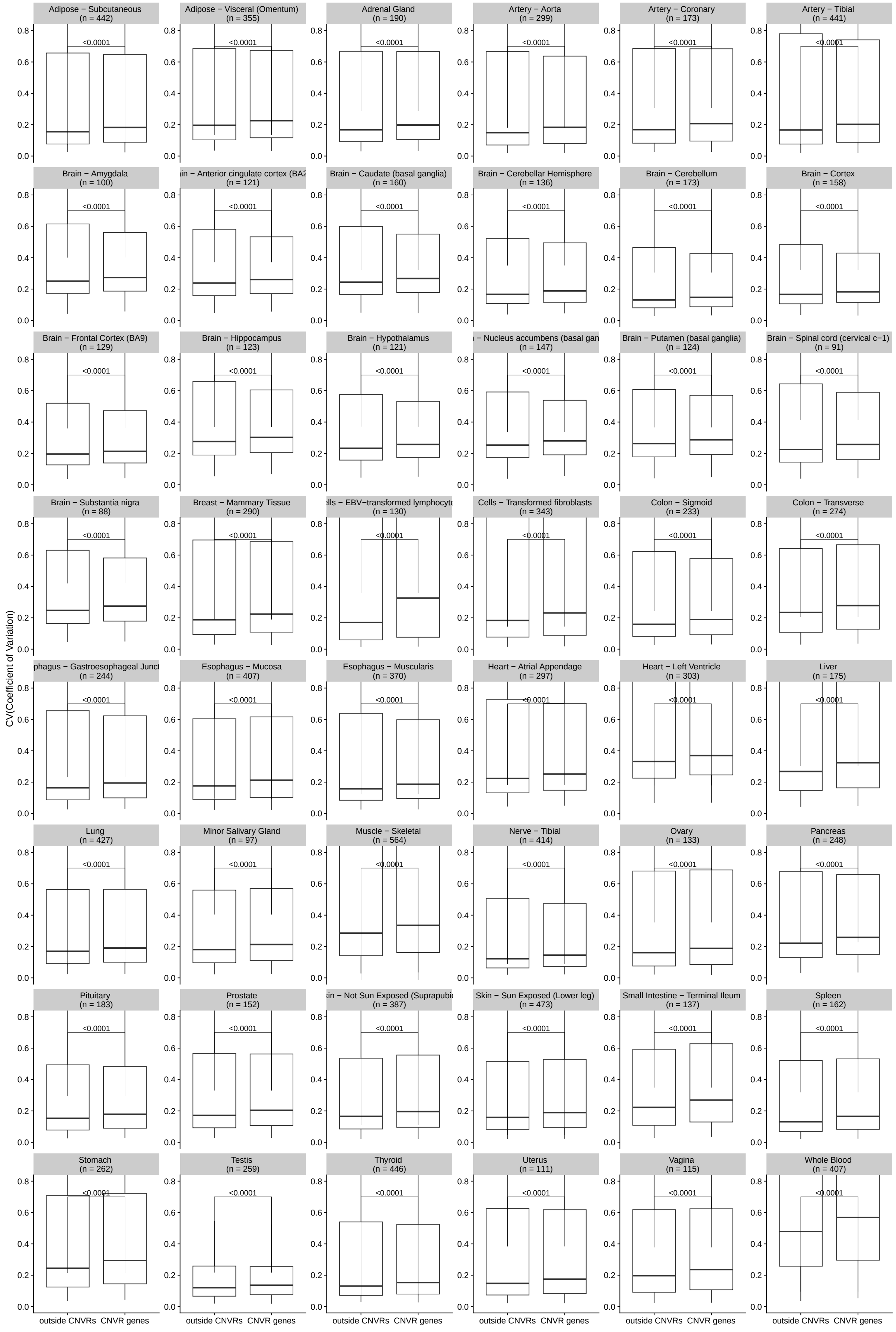

Figure S5. Coefficient of variation (CV) for genes outside CNVRs and CNVR genes

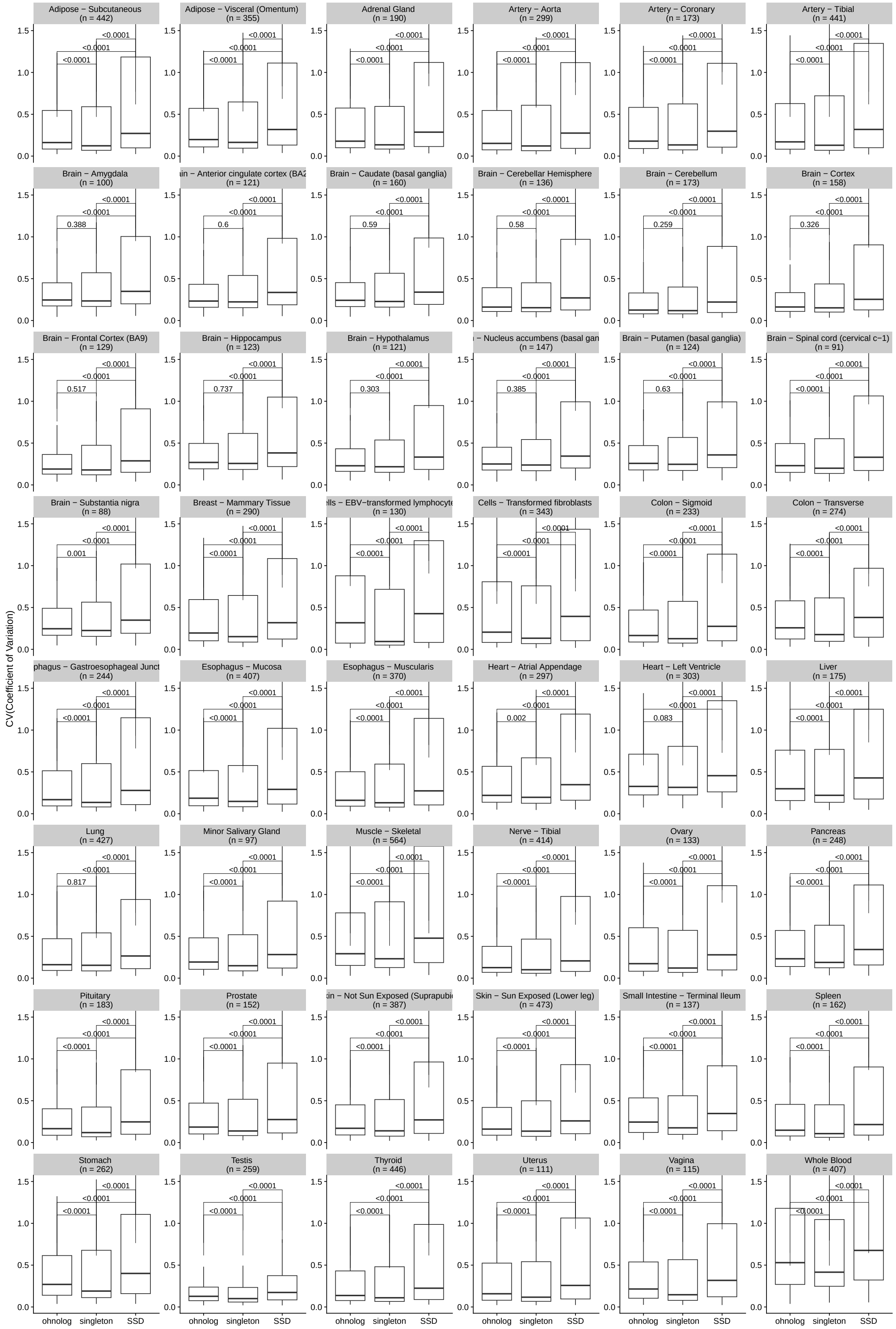

Figure S6. Coefficient of variation (CV) for ohnologs, singletons and SSDs

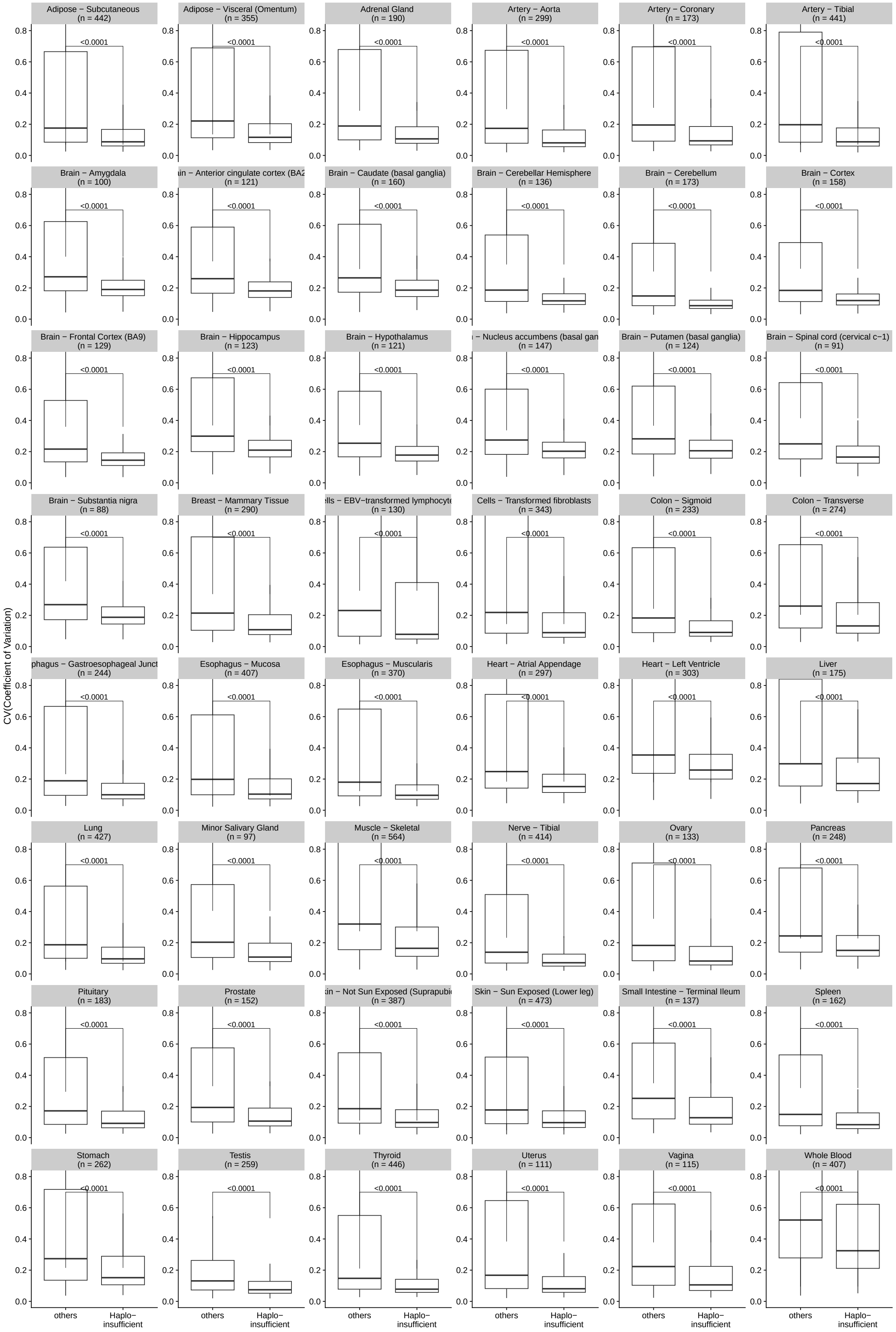

Figure S7. Coefficient of variation (CV) for haplo-sufficient and haplo-insufficient genes

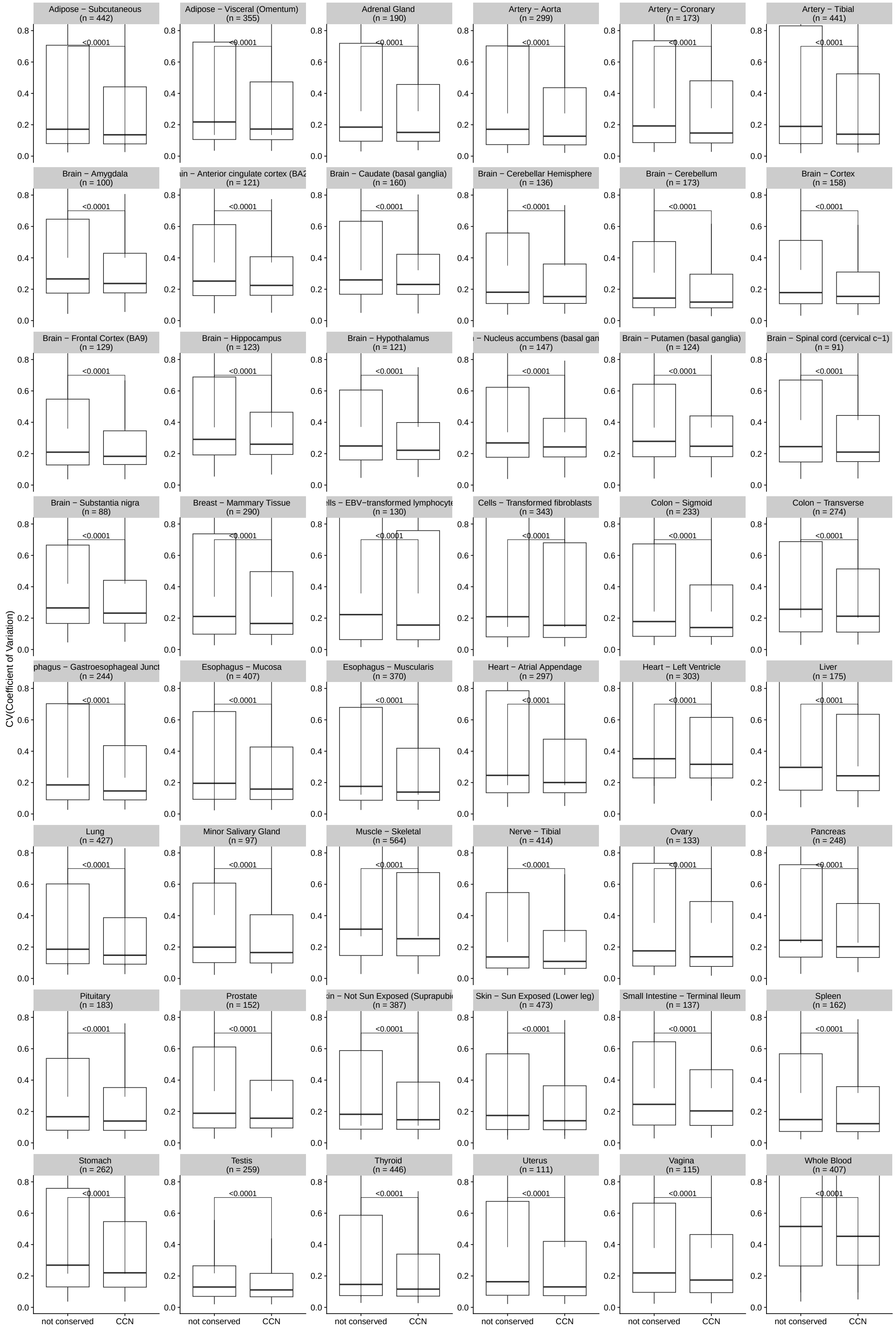

Figure S8. Coefficient of variation (CV) for conserved copy number and not conserved genes

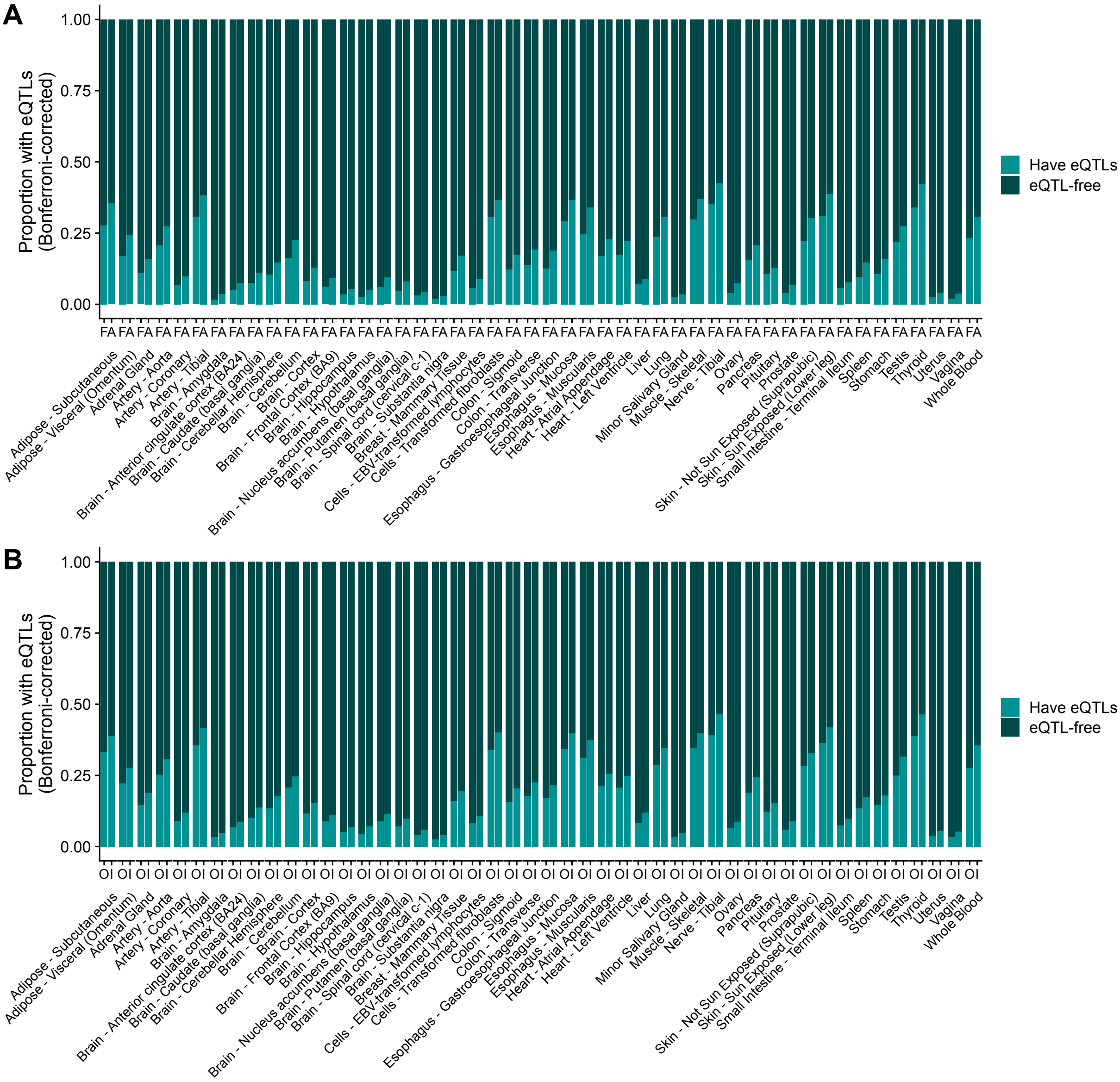

Figure S9. CNV-free genes (F) and genes outside CNVRs (O) have a lower proportion affected by eQTLs compared to CNV-affected genes (A) and genes in CNVRs (I) in each tissue.
A Bar chart for each of the 48 tissues analysed in the GTEx dataset showing the proportion of genes affected by eQTLs and grouped by whether the genes are affected by CNVs (A) or not affected by CNVs (F) in the Exome Aggregation Consortium (ExAC) dataset.
B Bar chart for each of the 48 tissues analysed in the GTEx dataset showing the proportion of genes affected by eQTLs and grouped by whether the genes are in CNVRs (I) or outside CNVRs (O) in the Zarrei et al. CNVR map dataset.

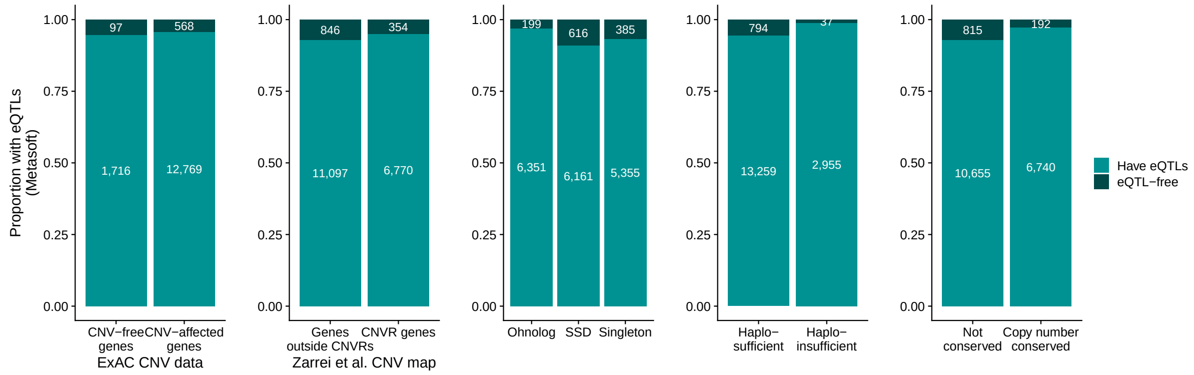

Figure S10. Proportion of genes affected by Metasoft eQTLs across different gene categories.
The proportion of genes (y-axis) within each gene category (x-axis) that are affected by Metasoft eQTLs. Gene categories include CNV-affected and CNV-free genes based on two CNV datasets (ExAC and Zarrei et al.), ohnologs (genes retained from whole-genome duplication), small-scale duplications (SSDs), singletons (genes without paralogs), haploinsufficient and haplosufficient genes, and genes with conserved copy number (CCN) and non-conserved copy number across mammals.

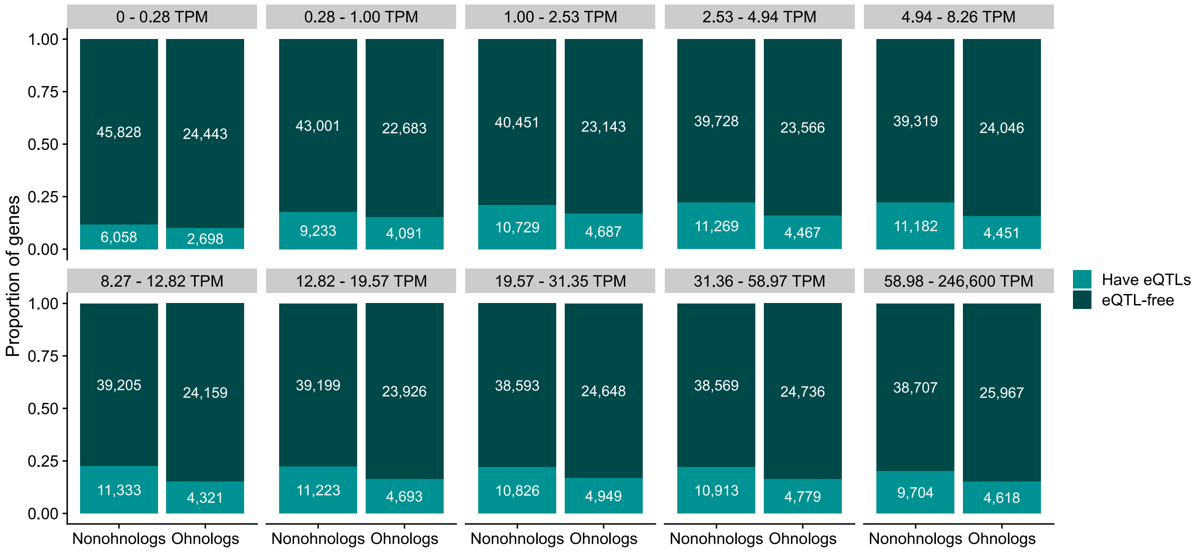

Figure S11. Proportion of ohnologs and non-ohnologs affected by eQTLs, grouped by median expression. The proportion of ohnologs and non-ohnologs affected by significant Bonferroni-corrected eQTLs. Genes are grouped into bins of roughly equal size based on their median expression level per tissue. The median expression value range in transcripts per million (TPM) for each bin is indicated above the bars.

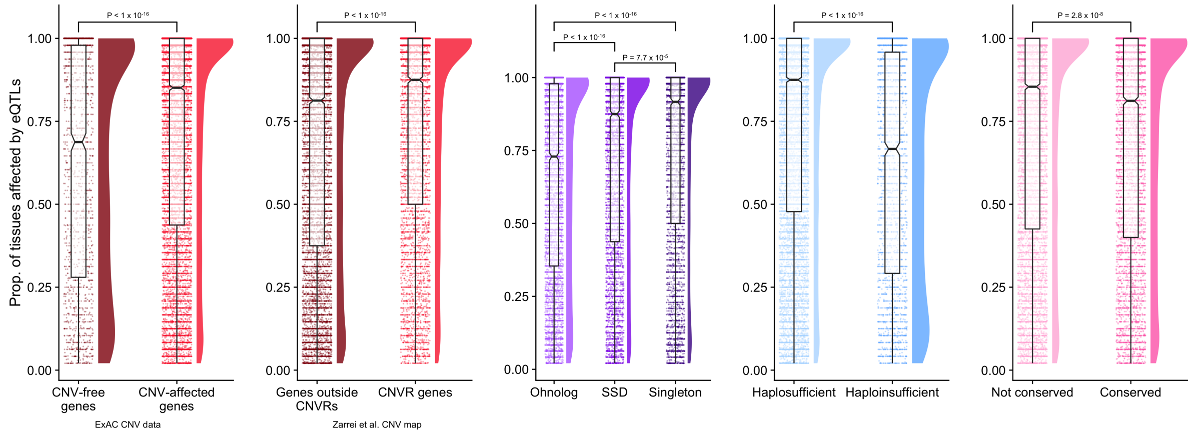

Figure S12. Proportion of expressed tissues that are affected by Metasoft eQTLs for eQTL-affected genes. Distributions of the proportion of expressed tissues that are affected by Metasoft eQTLs within each gene category. P-values above each group are for Mann-Whitney U tests and are Bonferroni-corrected.

purifying selection is acting to remove deleterious variants that conflict with expression constraints. A similar pattern may also arise if dosage-sensitive genes are in regions of reduced polymorphism and as a result are affected by fewer eQTLs. GTEx test SNPs within 1 megabase (Mb) of the transcription start site of each gene for significant SNP-gene eQTL associations. The number of SNPs tested per gene range from 119 to 28,260. We observe a significant difference in the number of SNPs tested by GTEx between ohnologs and singletons (median SNPs ohnologs: 7,251; singletons: 7,371, respectively, $P < 1 \times 10^{-16}$, Mann-Whitney U test) and between SSDs and singletons (median SNPs SSDs: 7,236, $P < 1 \times 10^{-16}$) but no difference between ohnologs and SSDs ($P = 1$).

Due to the differing amount of polymorphism around genes, we compared the proportion of SNPs tested that are found to be significant eQTLs for our gene groups rather than the absolute number of eQTLs (Figure S13). As the number of eQTLs for a given gene varies between tissues, a gene can be included multiple times for every tissue where it has at least one significant eQTL. However, the number of tested SNPs within 1 Mb of a gene is constant between tissues.

We find that both ExAC CNV-affected genes and Zarrei et al. CNVR genes have a higher proportion of SNPs that are significant eQTLs (median: 0.0053 and 0.0054) compared to CNV-free genes and genes outside CNVRs (median: 0.0039 and 0.0051,

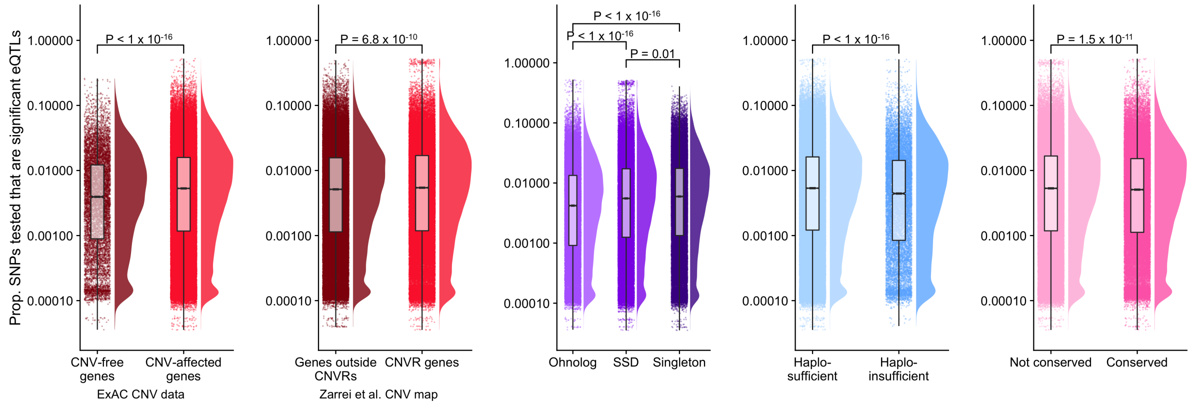

Figure S13. Proportion of tested SNPs that are significant Bonferroni-corrected eQTLs. For each gene/tissue combination with at least one significant Bonferroni-corrected eQTL, the proportion of GTEx-tested SNPs (within 1 Mb of the gene's transcription start site) that are found to be significant Bonferroni-corrected eQTLs (note the log10 scale on the y-axis). As the number of eQTLs varies between tissues, each gene can be included multiple times, for every tissue where it has at least one significant eQTL. P-values above each group are for Mann-Whitney U tests and are Bonferroni-corrected.

$P < 1 \times 10^{-16}$ and $P = 6.8 \times 10^{-10}$, Mann-Whitney U test, respectively). Ohnologs have a lower proportion of SNPs that are significant eQTLs (median: 0.0042) compared to SSDs and singletons (median: 0.0055 and 0.0060, $P < 1 \times 10^{-16}$ for both, Mann-Whitney U test). SSDs and singletons are relatively similar ($P = 0.01$). Haploinsufficicent and conserved copy number genes also have a lower proportion of SNPs that are significant eQTLs compared to haplosufficient genes and genes that do not have conserved copy number (Figure S13). Similar trends are found for Metasoft eQTLs (Figure S14).

Other mammalian genomes exhibit similar tissue-restricted expression changes

We wanted to explore whether the trends we observe for eQTLs affecting dosage-sensitive genes are unique to the human GTEx dataset and/or human genes, or are dosage-sensitive genes evolving by tissue-restricted expression changes observed elsewhere. To test this in another mammalian species, we employed a dataset of eQTLs from The Cattle Genotype-Tissue Expression atlas (cGTEx, Yao et al. 2022). This dataset was generated in a similar manner to the human GTEx project. Namely, cis-eQTL summary statistics

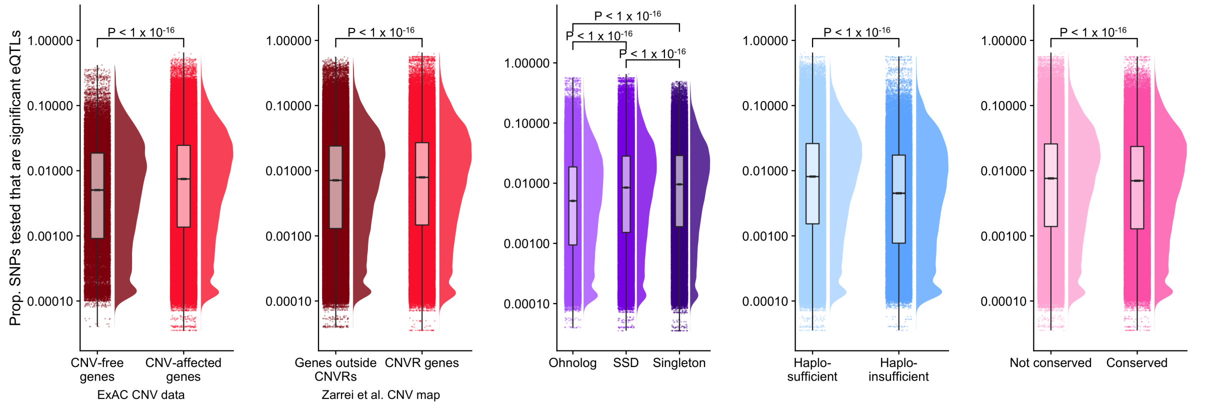

Figure S14. Proportion of tested SNPs that are significant Metasoft eQTLs. For each gene/tissue combination with at least one significant Metasoft eQTL, the proportion of GTEx-tested SNPs (within 1 Mb of the gene's transcription start site) that are found to be significant Metasoft eQTLs (note the log10 scale on the y-axis). As the number of eQTLs varies between tissues, each gene can be included multiple times, for every tissue where it has at least one significant eQTL. P-values above each group are for Mann-Whitney U tests and are Bonferroni-corrected.

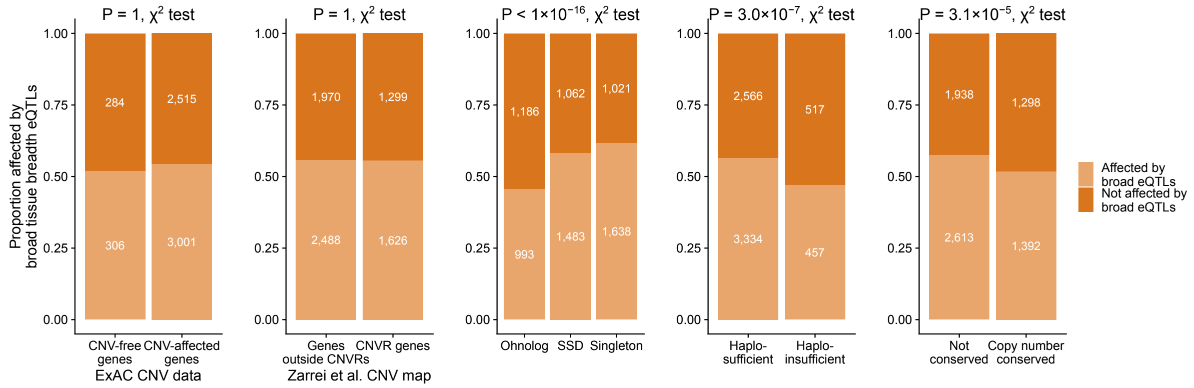

Figure S15. Proportion of genes affected by broad tissue breadth Metasoft eQTLs. Proportion of genes affected by broad tissue breadth Metasoft eQTLs (defined as affecting gene expression in 43 or more tissues). Bonferroni-corrected χ^2 test P-values are shown above each plot. Sample sizes are shown in white text for each group.

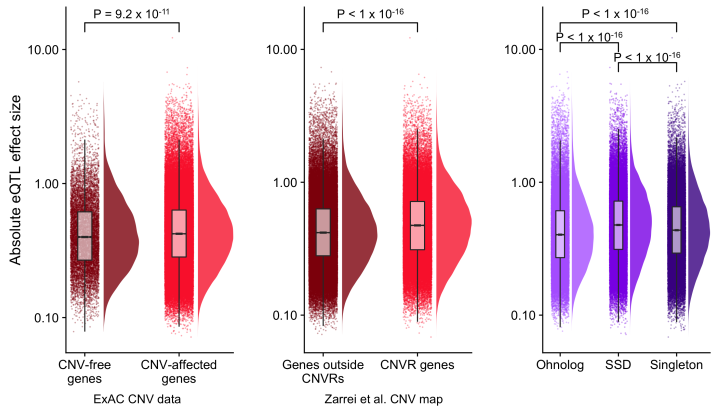

Figure S16. Absolute eQTL effect sizes for the most significant eQTL per gene/tissue in different gene groups. Distribution of absolute effect sizes (absolute value of the slope from the linear regression model) for the most significant Bonferroni-corrected eQTL associated with each gene in each tissue, stratified by gene category. Note the log10 y-axis scale and P-values above each group are for Mann-Whitney U tests and are Bonferroni-corrected.

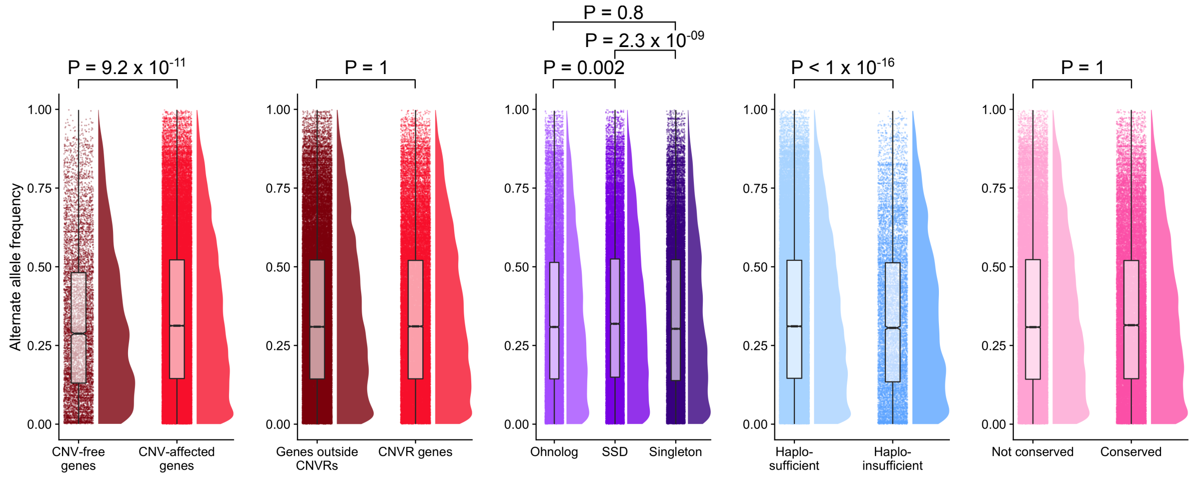

Figure S17. Allele frequency of SNPs associated with most significant eQTL per gene/tissue in different gene groups. Distribution of alternate allele frequencies (from the 1000 Genomes Project) for SNPs associated with the most significant Bonferroni-corrected eQTL for each gene in each tissue, stratified by gene category. P-values above each group are for Mann-Whitney U tests and are Bonferroni-corrected.

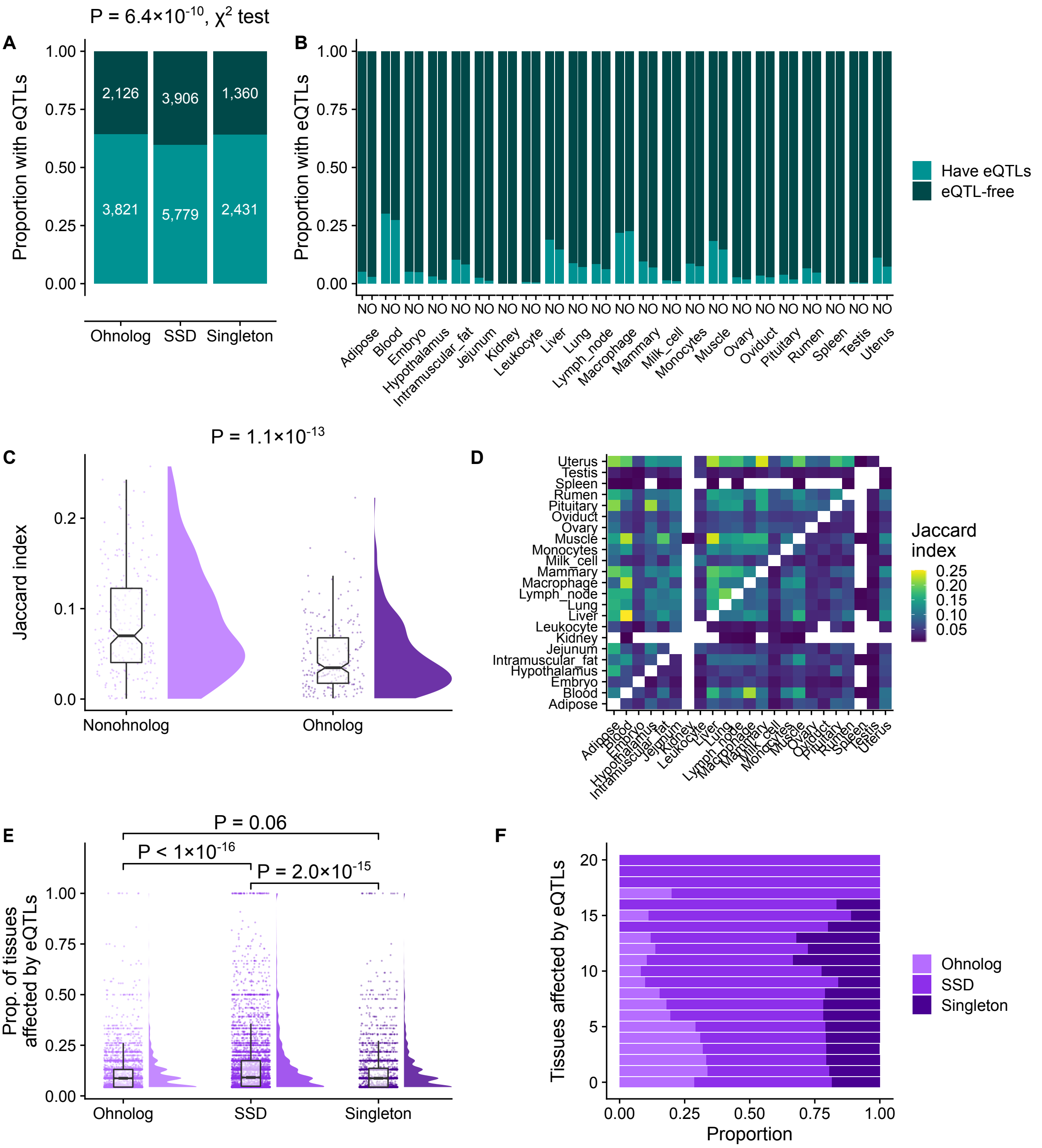

Figure S18. eQTL trends for ohnologs and non-ohnologs in the cow genome (cGTEx Data). Analysis of eQTL patterns in the cow genome using the cGTEx dataset, mirroring analyses performed on the human GTEx data. **A** Proportion of cow ohnologs, SSDs, and singletons affected by eQTLs in any tissue. P-value is for a Chi-squared test. **B** Proportion of cow ohnologs (O) and non-ohnologs (N) affected by eQTLs in each of the 23 tissues. **C** Distributions of pairwise Jaccard indices comparing eQTL-affected genes between tissues for ohnologs and non-ohnologs separately.P-value is from a Mann-Whitney U test. **D** Heatmap of pairwise Jaccard indices between eQTL-affected genes in individual tissues. The upper triangle shows overlap for non-ohnologs; the lower triangle shows overlap for ohnologs. **E** For each eQTL-affected gene, the proportion of tissues where the gene is expressed that are affected by eQTLs. P-values from Mann-Whitney U tests. **F** Proportion of ohnologs, SSDs, and singletons categorised by the number of tissues affected by eQTLs.

were downloaded from The Cattle Genotype-Tissue Expression atlas (cGTEx) from `https://cgtex.roslin.ed.ac.uk/` (Yao et al. 2022). These were processed using the cGTEX script

4_cis-eQTL_p-value.nominal.correction_basePermutation.r to obtain a list of permutation corrected eQTLs that were subsequently Bonferroni-corrected for multiple testing for the number tissues tested.

We find that ohnologs in the cow genome are enriched for being affected by eQTLs when all tissues are considered together, similar to what we observe of human ohnologs (Figure S18A). We observe that 64.3% of cow ohnologs are affected by eQTLs in at least one of their expressed tissues compared to 59.7% of SSDs and 64.1% of singletons ($P = 6.4 \times 10^{-10}$, χ^2 test). As observed in Figure **??**A, human ohnologs are enriched, and SSDs and singletons are depleted (standardised residuals of ohnologs: 7.9, SSDs: -4.8, singletons: -3.2), while in cow, singletons are also enriched (standardised residuals of ohnologs: 4.4, SSDs: -6.5, singletons: 3.1).

Considering individual tissues, ohnologs in the cow genome are less affected by eQTLs in 20 of 23 tissues/cell types (Figure S18B), with kidney, spleen and macrophage being the three tissues that differ from the trend. However, kidney and spleen have fewer than 20 eQTL-affected genes each so sample size might be a factor affecting the results. The remaining unusual tissue/cell type is macrophage (22.8% of ohnologs affected by eQTLs compared to 22.0% of non-ohnologs). When eQTL-affected genes are compared pairwise between tissues using the Jaccard index, eQTL-affected ohnologs are less shared between tissues compared to eQTL-affected non-ohnologs (Figure S18C and D), as was seen for the human data. We find significantly lower similarity among eQTL-affected ohnologs compared to eQTL-affected non-ohnologs (median Jaccard index of 220 tissue comparisons of eQTL-affected ohnologs: 0.04 vs. 0.07 for non-ohnologs; $P 1.1 \times 10^{-13}$, Mann-Whitney U test).

As in the human dataset, we observe that ohnologs in the cow genome have a lower proportion of tissues affected by eQTLs compared to SSDs (median proportion of expressed

tissues affected by eQTLs: 8.7% and 9.1%, respectively; $P < 1 \times 10^{-16}$, Mann-Whitney U test; Figure S18E and F). However, contrasting with the human data we observe that singletons in the cow genome have a lower proportion compared to SSDs but are not different from ohnologs (median proportion of expressed tissues affected by eQTLs: 8.7% and 9.1%, respectively; $P < 1 \times 10^{-16}$). In cow, singletons are not significantly different from ohnologs whereas in human, SSDs and singletons are similar and different from ohnologs.

References

Yao, Yuelin et al. (Aug. 2022). "Comparative transcriptome in large-scale human and cattle populations". en. *Genome Biology* 23.1, p. 176. DOI: 10.1186/s13059-022-02745-4.

Zarrei, Mehdi et al. (Feb. 2015). "A copy number variation map of the human genome". *Nature Reviews Genetics* 16.3, pp. 172–183. DOI: 10.1038/nrg3871.
